# Water intake regulates mucosal immunity in rat jejunal villi via IL‐1β, IL‐6, and IL‐10

**DOI:** 10.14814/phy2.70891

**Published:** 2026-04-29

**Authors:** Norika Kuneshita, Yuki Sakai, Moyuru Hayashi, Tomomi Watanabe‐Asaka, Daisuke Maejima, Yoshiko Kawai, Toshio Ohhashi

**Affiliations:** ^1^ Department of Innovation of Medical and Health Sciences Research Shinshu University School of Medicine Matsumoto Japan; ^2^ Department of Dentistry and Oral Surgery Shinshu University School of Medicine Matsumoto Japan; ^3^ Division of Physiology Tohoku Medical and Pharmaceutical University, Faculty of Medicine Sendai Japan

**Keywords:** clodronate, cytokines, jejunum, mucosal immunity, MyD88, water intake

## Abstract

We had demonstrated that the intragastric administration of distilled water increased male rat jejunal lymph flow and interleukin‐22 levels in the lymph, resulting in the promotion of innate immunity. However, there is no information concerning whether the water intake regulates mucosal immunity in the jejunum of male rats. Based on the evidence, we investigated the effects of distilled water intake on gut immunity in the rat jejunum. Water intake significantly stimulated the release of IL‐1β and IL‐6 in mesenteric lymph. However, the concentration of IL‐10 was not changed by water intake. Pretreatment with clodronate significantly decreased the lymph flow at 120–180 min after the intake. Clodronate significantly reduced water intake‐mediated release of IL‐1β and decreased, but not significantly, the release of IL‐6. MyD88 inhibitor significantly decreased water intake‐mediated release of IL‐1β and IL‐6. However, clodronate and MyD88 inhibitor did not significantly change IL‐10 in the lymph. The expression levels of the macrophage markers CD68, F4/80, and CD169 in the jejunal villi significantly decreased due to pretreatment with clodronate. These findings suggest that water intake acts as a trigger for starting mucosal immunity in the jejunum via macrophage and MyD88‐mediated activation of the release of IL‐1β and IL‐6.

## 1 INTRODUCTION

Water intake plays a crucial role in maintaining health care for humans, which is the Japanese custom in long periods. Thus, the traditional Japanese health care system recommends that a suitable volume of water be consumed every day (Wang & Li, 2020). However, the detailed mechanisms of water intake‐mediated health care were still unknown. Thus, to clarify the mechanisms of water intake‐mediated health care, we have conducted in vivo animal experiments (Hayashi et al., 2021; Nagashio et al., 2019) to investigate the effects of water intake on jejunal‐originated lymph flow and innate lymphoid cell 3 (ILC‐3)‐secreted interleukin‐22 (IL‐22) in rat lymph and revealed the importance of water in the jejunal microcirculation through the transport of consumed water into the jejunum‐originated mesenteric lymph vessels (Hayashi et al., 2021; Nagashio et al., 2019). Furthermore, water intake accelerated the transfer of IL‐22 to the mesenteric lymph, which may contribute, in part, to maintaining and promoting innate immunity. In addition, water intake accelerated the serotonin production and release in rat jejunum. The intravenous administration of serotonin in rats significantly increased mesenteric lymph flow and the concentration of IL‐22 in the lymph (Kajihara et al., 2021).

Moreover, we have suggested that a strong bulk flow‐dependent mechanical force resulting from water intake accelerates ATP release from myofibroblast in rat jejunal villi. The released ATP increases the IL‐22 production in the jejunum (Hayashi et al., 2021). However, no or few studies on the water intake‐mediated regulation of mucosal immunity in the jejunum with inflammatory and anti‐inflammatory cytokines exist. In contrast, cocoa butter intake regulates gut immunity through the release and transport of IL‐1β, IL‐6, and IL‐10 into mesenteric lymph vessels in a negative feedback system (Arai et al., 2023).

Based on this evidence, we aimed to investigate whether water intake plays crucial roles in the regulation of mucosal immunity in rat jejunal villi with IL‐1β, IL‐6, and IL‐10. In addition, to clarify the relationship between the regulation of mucosal immunity and the roles of macrophages in jejunal villi, the effects of clodronate‐containing liposomes, which are macrophage depletion substances, on water intake‐dependent mucosal immunity were evaluated. To confirm the effects of clodronate on macrophages in jejunal villi, we investigated changes in the immunoreactivities of the macrophage markers, CD68, F4/80, and CD169, in the jejunum. Finally, the effects of clodronate and an inhibitor of myeloid differentiation primary response gene (MyD)88, which is a key molecule required for signaling mediated by Toll‐like receptors (TLR) (Choy et al., 2020; Iimuro et al., 2007; Latz et al., 2013; Saraiva & O'Garra, 2010) in macrophages, on the water intake‐mediated regulation of mucosal immunity were evaluated.

## 2 MATERIALS AND METHODS

### 2.1 In vivo rat experiments

All experimental protocols in this study were approved by the Institutional Animal Care Use Committee of Shinshu University (No. 023017, 1st April, 2019) and followed the ARRIVE Guideline.

Forty male Sprague–Dawley rats (10–12‐weeks‐old; Japan SLC, Tokyo, Japan) were fed a standard pellet diet (MF, Oriental Yeast, Tokyo, Japan) and provided water ad libitum. The animals were fasted and inhibited drinking water overnight to reduce the effect of feeding on mesenteric lymph flow before the experiments. The temperature and moisture of housing condition for rats were maintained at 26°C–28°Cand 40%–50% using air conditioners, respectively. The rats were anesthetized with 2.0%–3.0% isoflurane (under N_2_O + 100% O_2_ inhalation at a 2:1 ratio; Dainippon Sumitomo Pharma, Tokyo, Japan) and then placed on an operating table in the supine position. A catheter was inserted into the femoral vein to inject the physiological saline solution (PSS; Otsuka Pharma, Tokyo, Japan) used in the experiments. PSS (2 ~ 3 mL/ min) was intravenously administered to maintain normal physiological conditions in the rats before the experiments began. The minimized volume of PSS used was also decided to keep the venous administration rout and compensate the body fluid loosed during the operation. To minimize the hemodynamic changes in the jejunal microcirculation, intravenous infusion of PSS was stopped during the experiments (Hayashi et al., 2021; Nagashio et al., 2019). To collect the lymph from the jejunal‐originated mesenteric lymph vessels, the abdomen was opened by cutting along the midline, and mesenteric adipose and connective tissues were removed to expose the mesenteric lymph node located outside the jejunum and the efferent lymph vessel. A heparinized small polyethylene catheter (0.5–0.6 mm) was inserted centrifugally into an efferent lymph vessel (Hayashi et al., 2021; Nagashio et al., 2019).

To evaluate effects of the intragastric administration of 3.0 mL distilled water from the oral space on the jejunal‐originated mesenteric lymph flow and the levels of cytokines, IL‐1β, IL‐6, and IL‐10 in the lymph, the lymph was collected over a set period (60 min), and its lymph volume was measured. We confirmed histologically that the administration produced no inflammation in the jejunum villi. We previously measured time‐dependent changes in rat jejunal‐origin mesenteric lymph volume with the intragastric administration of distilled water and then determined the maximal lymph volume during the first 60 min after administration (Kajihara et al., 2021). In the control experiment, we conducted the anesthetized rats inserted same‐typed intragastric tube without administration of distilled water.

Distilled water was selected because an earlier study (Simmonds, 1955) demonstrated that the injection of 5 mL of distilled water, but not PSS, injected into the rat stomach rapidly increased the degree of lymph flow through the thoracic duct, compared with the administration of PSS (Hayashi et al., 2021; Nagashio et al., 2019). In the preliminary experiments, we confirmed that compared with the administration of distilled the intragastric administration of an isotonic saline solution accelerated the rate of rat jejunal‐originated lymph flow in compared with the administration of distilled water (3, 4). Moreover, the distilled water was selected because it has no ionic substances that interfere with the effects of ionic osmotic pressure on the absorption of water in the jejunum (Hayashi et al., 2021; Nagashio et al., 2019).

To investigate the effects of macrophages in the jejunal villi on the water intake‐mediated changes in the levels of IL‐1β, IL‐6, and IL‐10 in the lymph, we investigated the effects of 24–48 h of intraperitoneal clodronate‐containing liposomes in conscious rats (catalog no 16001004, Sigma‐Aldrich, USA), a macrophage depletion reagent, on the water intake‐mediated changes in the concentrations of the cytokines transported into the mesenteric lymph. To confirm the effect of clodronate on the regulation of mucosal immunity, we also investigated the effects of intravenous administration of MyD88, a key adaptive poteen associated with IL‐1β and almost Toll‐like receptors, homodimerization inhibitory peptide inhibitor (catalog no NBP2‐29328, Novus Biological, USA) homodimerization in, an inhibitor of MyD88 on the water intake‐mediated changes in the concentrations of IL‐1β, IL‐6, and IL‐10 were examined.

### 2.2 Measurements of IL‐1β, IL‐6, and IL‐10 levels in the mesenteric lymph

First, the obtained mesenteric lymph was centrifuged, and the concentrations of IL‐1β, IL‐6, and IL‐10 in the supernatants were measured. The concentrations of cytokines in the lymph were measured using enzyme‐linked immunosorbent assay (ELISA) kits: a rat IL‐1β ELISA quantitative kit (catalog no. RLB00; R&D Systems, Minneapolis, MN, USA), an IL‐6 ELISA kit (catalog no. R6000B; R&D Systems, Minneapolis, MN, USA), and a mouse/rat IL‐10 ELISA kit (catalog no. KE20003; Rosemont, IL, USA) (Arai et al., 2023).

### 2.3 Immunohistochemistry

To evaluate the effects of the intraperitoneal administration of clodronate‐containing liposomes (20 mg) on the distribution of macrophages in the jejunal villi, the immunoreactivities of selective markers of the macrophages in the gut, CD68, F4/80, and CD169, in the lamina propria of jejunal villi were investigated. The jejunum, which was loaded with or without clodronate for 24 h, was rapidly isolated. The rats were anesthetized by intraperitoneal injection of pentobarbital sodium (10 mg/kg iv; catalog no. 76‐74‐4 Sigma‐Aldrich, USA), after which the small intestines and mesenteric lymph nodes were isolated. Finally, the rats were killed by bleeding via cardiac puncture. The upper and lower parts of both the jejunum and ileum were isolated in quarters along the length of the small intestine, washed with excess PSS, and fixed with 4% paraformaldehyde in PBS overnight. The fixed samples were sectioned, and the slices were washed three times with PBS and incubated for 2 h at room temperature with primary polyclonal antibodies to FITC‐labeled CD68 (catalog no. bs‐06449R, Bioss, Boston, USA), F4/80 (catalog no. 28463‐1‐AP, Proteintech, Rosemont, USA), or CD169 (catalog no. bs‐10751R, Bioss, Boston, USA), all diluted to 1:5000. After being washed three times in PBS, the tissue slices were mounted with Pro‐Long Gold antifade reagent and 4′‐6‐diamidino‐2–2‐phenylindole (DAPI, catalog no. P36935; Invitrogen, Waltham, USA) to counterstain the cell nuclei. The slices were examined under a fluorescence microscope (×2 ~ 40 objective lenses, BZ9000, KEYENSE, Osaka, Japan) and photographed (Hayashi et al., 2021).

### 2.4 Density measurement

To quantify the immunoreactivity data obtained at the same brightness, high‐resolution digital photomicrographs were processed using the Scion image analysis program (Kawai et al., 2010). The constant area of each photomicrograph was outlined on a grayscale image at the same density and processed for density measurements. The 10 fields examined per 4 tissue samples per each rat. The results are expressed in arbitrary units (mean density per pixel).

### 2.5 Drugs

All the chemicals were obtained from Wako (Tokyo, Japan). Heparin sulfate was purchased from Mochida Pharmaceutical Co. (Tokyo, Japan). Clodronate‐containing liposomes (catalog no 16001004, Sigma‐Aldrich, USA) and an inhibitor of MyD88 (catalog no 2‐29328 Novus Bio, USA) are purchased. Drug concentrations were described as the final concentrations in PBS.

### 2.6 Statistical analysis

All results are expressed as the mean ± standard deviation (SD). Statistical analyses were performed using Student's *t*‐test for paired or unpaired results or one‐way ANOVA, followed by Bonferroni correction, as appropriate. Differences were considered statistically significant at *p* < 0.05.

## 3 RESULTS

### 3.1 Water intake increased IL‐1β, IL‐6 and IL‐10 levels in rat mesenteric lymph

**FIGURE 1:**
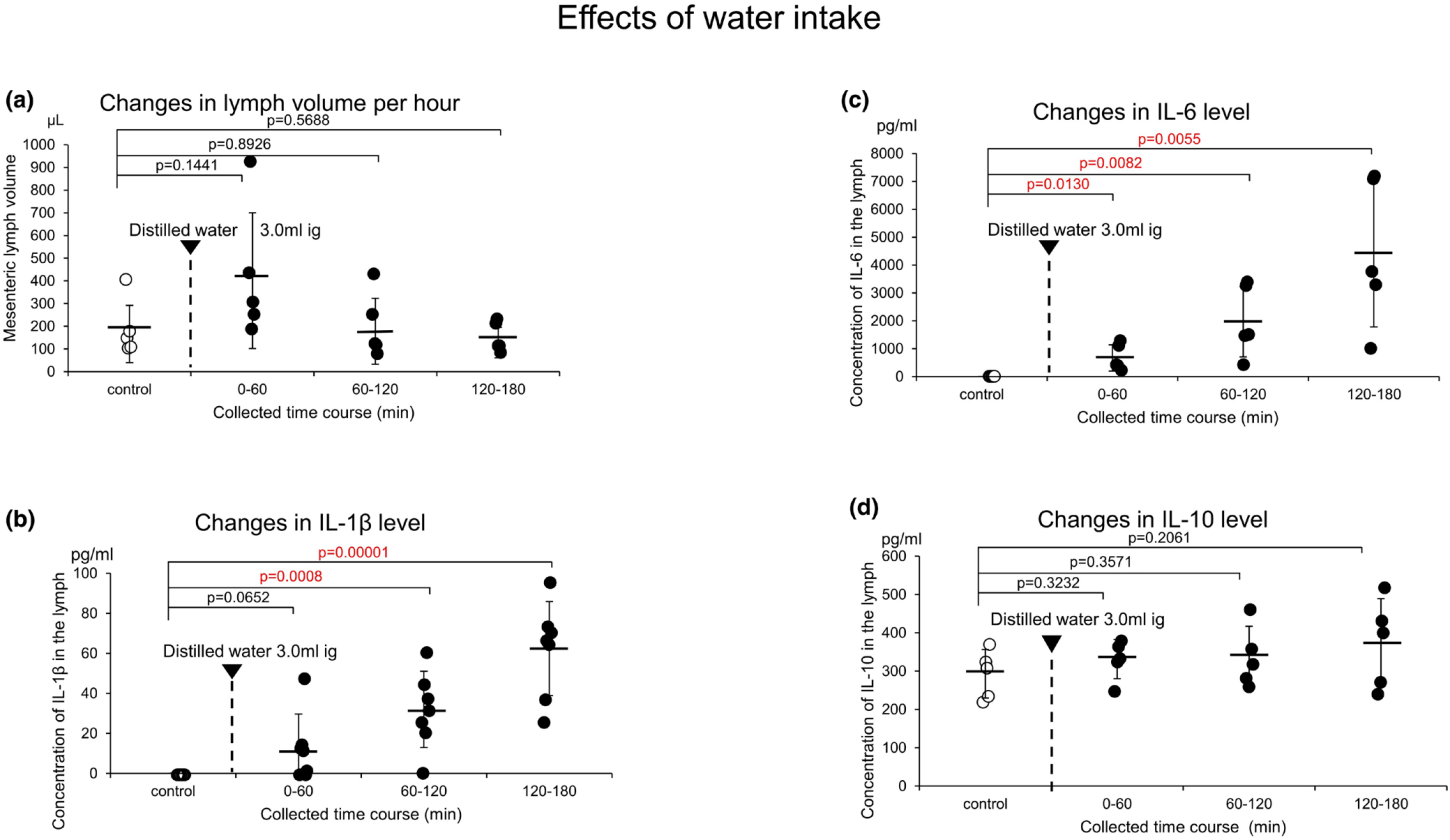
(a) Effects of water intake on changes in lymph volume collected over set intervals of 60 min in rat jejunum‐originated lymph vessels. (b) Effects of water intake on changes in the concentration of IL‐1β in the lymph. (c) Effects of water intake on changes in the concentration of IL‐6 in the lymph. (d) Effects of water intake on changes in the concentration of IL‐10 in the lymph. The open column, control; the black column, water intake. The error bars represent SDs.

Figure 1a shows the effects of the intragastric administration of 3.0 mL of distilled water on the lymph volume collected over set intervals of 60 min from rat jejunum‐derived lymph vessels (the control 166.0 ± 126.2 μL/60 min; 0–60 min 401.0 ± 299.0 μL/60 min vs. the control, *p* = 0.1441 *n* = 5; 60–120 min 178.0 ± 145.5 μL/60 min vs. the control, *p* = 0.8926 *n* = 5; 120–180 min 128.0 ± 67.2 μL/60 min vs. the control, *p* = 0.5688 *n* = 5). Water intake tended to slightly increase the lymph volume from 0 to 60 min, but the difference was not significant.

The concentration of the cytokine IL‐1β increased in a time‐dependent manner in the lymph collected over intervals of 60 min after water intake (3.0 mL) (Figure 1b). The following data were obtained (control 0.0 ± 0.0 pg/mL; 0–60 min 13.0 ± 16.8 pg/mL vs. the control, *p* = 0.0652 *n* = 5; 60–120 min 32.0 ± 19.0 pg/mL vs. control, *p* = 0.0008 *n* = 5; 120–180 min 62.0 ± 23.5 pg/mL, *p* = 0.00001 vs. control *n* = 5). Thus, water intake resulted in a significant increase in IL‐1β levels from 60 to 180 min.

High concentrations of cytokine IL‐6 were released in a time‐dependent manner into mesenteric lymph following water intake. The concentrations of IL‐6 in the lymph collected over intervals of 60 min are shown in Figure 1c (0.0 ± 0.0 pg/mL; 0–60 min 670.0 ± 470.7 pg/mL vs. the control, *p* = 0.0130 *n* = 5; 60–120 min 1971.0 ± 1263.2 pg/mL vs. the control, *p* = 0.0082 *n* = 5; 120–180 min 4395.0 ± 1168.9 pg/mL vs. the control, *p* = 0.0055 *n* = 5). Thus, the water intake resulted in a significant increase in IL‐6 concentration from 0 to 180 min.

The anti‐inflammatory cytokine IL‐10 was also confirmed in the lymph collected over intervals of 60 min. However, a marked increase in the level of IL‐10 in the lymph was not detected in response to water intake. The summarized data (293.0 ± 63.3 pg/m; 0–60 min 331.0 ± 51.2 pg/mL vs. the control, *p* = 0.3232 *n* = 5; 60–120 min 337.0 ± 79.6 pg/mL vs. the control, *p* = 0.3571 *n* = 5; 120–180 min 374.0 ± 115.3 pg/mL vs. the control, *p* = 0.2061 *n* = 5). These results indicate that water intake did not significantly change IL‐10 levels in the mesenteric lymph.

### 3.2 Pretreatment with clodronate tended to reduce the lymph volume from 0 to 120 min and then decreased it significantly it from 120 to 180 min

**FIGURE 2:**
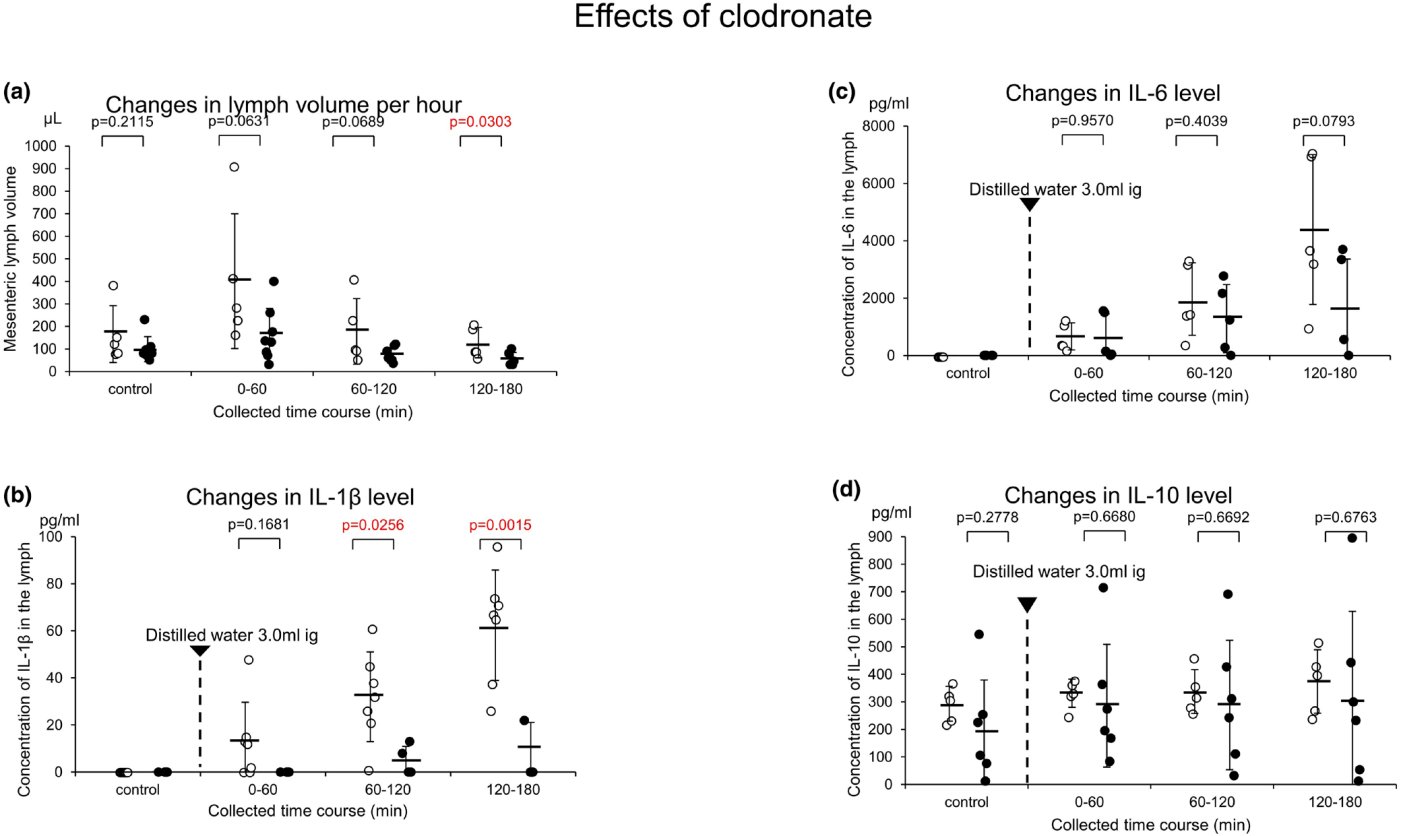
(a) Effects of water intake on changes in lymph volume collected over set intervals of 60 min in rat jejunum‐derived lymph vessels in the absence (white column) and presence of clodronate (oblique line column). (b) Effects of water intake without (white column) and with clodronate (oblique line column) on changes in the concentration of IL‐1β in the lymph. Effects of water intake without (white column) and with clodronate (oblique line column) on changes in the concentration of IL‐6 in the lymph. Effects of water intake without (white column) and with clodronate (oblique line column) on changes in the concentration of IL‐10 in the lymph. The error bars represent SDs.

Pretreatment with clodronate tended to reduce the lymph volume collected over intervals of 60 min from the jejunum‐derived lymph volume (Figure 2a; control without clodronate 166.0 ± 126.2μL/60 min vs. control with clodronate 99.4 ± 55.6 μL/60 min, *p* = 0.2115 *n* = 5; 0–60 min water intake without clodronate 401.0 ± 299.0 μ L/60 min vs. 0–60 min water intake with clodronate 160.6 ± 119.5 μL/60 min, *p* = 0.0631 *n* = 5; 60–120 min water intake without clodronate 178.0 ± 145.5 μL/60 min vs. 60–120 min water intake with clodronate 73.1 ± 31.6 μ L/60 min, *p* = 0.0669 *n* = 5; 120–180 min water intake without clodronate 128.0 ± 67.2 μ L/60 min vs. 120–180 min with clodronate 58.6 ± 25.9 μL/60 min, *p* = 0.0303 *n* = 5). Thus, treatment with clodronate resulted in a significant decrease in the lymph volume from 120 to 180 min.

### 3.3 Pretreatment with clodronate markedly reduced the water intake‐mediated release of IL‐1β and IL‐6, but did not affect the concentration of IL‐10 in the lymph

Pretreatment with clodronate significantly decreased the water intake‐mediated release of IL‐1β (Figure 2b, IL‐1β; the control without clodronate 0.0 ± 0.0 pg/mL vs. the control with clodronate 0.0 ± 0.0 pg/mL, NS *n* = 5; 0–60 min water intake without clodronate 13.0 ± 16.8 pg/mL vs. 0–60 min water intake with clodronate 0.0 ± 0.0 pg/mL, *p* = 0.1681 *n* = 5; 60–120 min water intake without clodronate 32.0 ± 19.0 pg/mL vs. 60–120 min water intake with clodronate 5.3 ± 5.7 pg/mL, *p* = 0.0256 *n* = 5; 120–180 min water intake without clodronate 62. 0 ± 23.5 pg/mL vs. 60–120 min water intake with clodronate 5.5 ± 15.6 pg/mL, *p* = 0.0015 *n* = 5).

Clodronate also markedly reduced, but not significantly, the water intake‐mediated release of IL‐6 (Figure 2c, the control water intake without clodronate 0.0 ± 0.0 μL/60 min vs. the control water intake with clodronate 0.0 ± 0.0 pg/mL, NS *n* = 5; 0–60 min water intake without clodronate 670.0 ± 470.7 pg/mL vs. 0–60 min water intake with clodronate 646.6 ± 805.5 pg/mL, *p* = 0.9570 *n* = 5; 60–120 min water intake without clodronate 1971.0 ± 1263.2 pg/mL vs. 60–120 min water intake with clodronate 1288.0 ± 1188.3 pg/mL, *p* = 0.4039 *n* = 5; IL‐6; 120–180 min water intake without clodronate 4395. 0 ± 2613.0 pg/mL vs. 120–180 min water intake with clodronate 1519.2 ± 1845.9 pg/mL, *p* = 0.0793 *n* = 5).

Pretreatment with clodronate caused no significant change in the water intake‐mediated release of IL‐10 in the lymph (Figure 2d, control water intake without clodronate 293.0 ± 63.3 pg/mL vs. control water intake with clodronate 189.8 ± 189.7 pg/mL, *p* = 0.2778; 0–60 min water intake without clodronate 331.0 ± 51.2 pg/mL vs. control water intake with clodronate 285.8 ± 230.0 pg/mL, *p* = 0.6680 *n* = 5; 60–120 min water intake without clodronate 337.0 ± 79.6 pg/mL vs. 60–120 min water intake with clodronate 288.5 ± 235.2 pg/mL, *p* = 0.6692 *n* = 5; 120–180 min water intake without clodronate 374.0 ± 115.3 pg/mL vs. 120–180 min water intake with clodronate 308.5 ± 320.2 pg/mL, *p* = 0.6763 *n* = 5).

### 3.4 Pretreatment with clodronate significantly decreased the immunoreactivities of CD68, F4/80, and CD169

**FIGURE 3:**
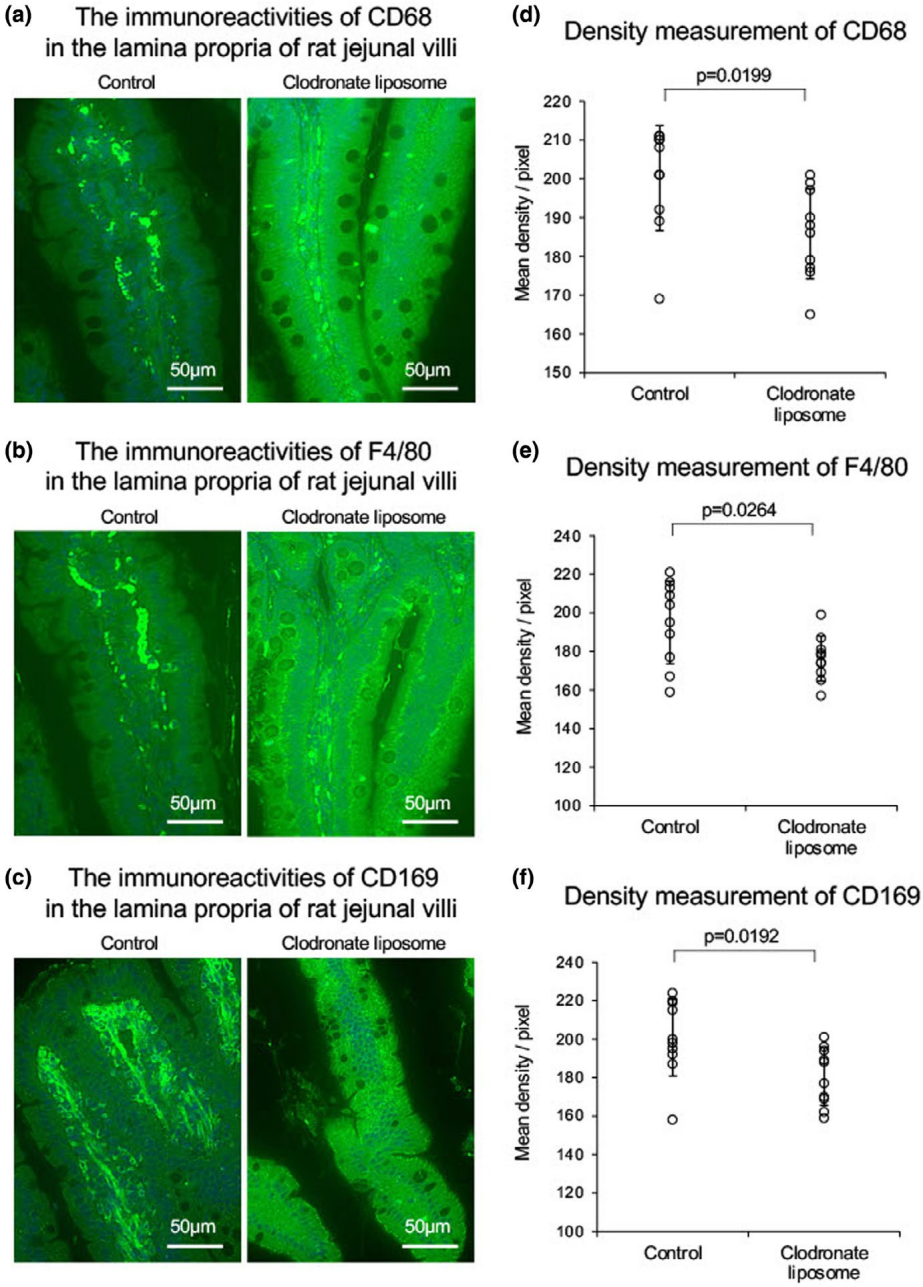
(a) Representative immunoreactivities CD68 of a marker of macrophage in the lamina propria of rat jejunal villi without (control) and with the clodronate treatment. (b) Representative immunoreactivities of F4/80, a marker of macrophage in the lamina propria of rat jejunal villi without (control) and with the clodronate treatment. (c) Representative immunoreactivities of CD169, a marker of macrophage in the lamina propria of rat jejunal villi without (control) and with the clodronate treatment. (d–f) Summarized data of density analyses of CD69, F4/80, and CD169, respectively. The error bars represent SDs.

Representative photomicrographs of CD68‐positive cells in the jejunal villi (Control) and the jejunal villi pretreated with clodronate (20 mg, 24 h, Clodronate liposome) are shown in Figure 3a. In the Control, marked CD68 immunoreactivity was observed in the lamina propria of jejunal villi. Compared with the Control, the CD68 immunoreactivity decreased in the preparation pretreated with clodronate. Consistent with these findings, the F4/80 and CD169 immunoreactivities in the lamina propria of jejunal villi was similarly reduced by the pretreatment with clodronate (Figure 3b,c). The CD68, F 4/80 and CD169 immunoreactivities in the lamina propria of jejunal villi were quantitatively analyzed using density measurement. The data were summarized in Figure 3d–f. The expression level of CD68 (cont: 200.2 ± 13.6 vs. clodronate 185.8 ± 11.6, *p* = 0.020 *n* = 10), F4/80 (cont 195.0 ± 21.5 vs. clodronate 176.3 ± 11.7, *p* = 0.026 *n* = 10) and CD169 (cont: 200.8 ± 19.9 vs. clodronate 180.5 ± 15.0, *p* = 0.019 *n* = 10) in immunohistochemistry images were significantly reduced by the pretreatment with clodronate. No or little inflammation was confirmed with no invasion of neutrophil leucocyte and no bleeding in the jejunum villi.

### 3.5 Pretreatment with a MyD88 inhibitor tended to reduce, but not significant, the lymph volume

**FIGURE 4:**
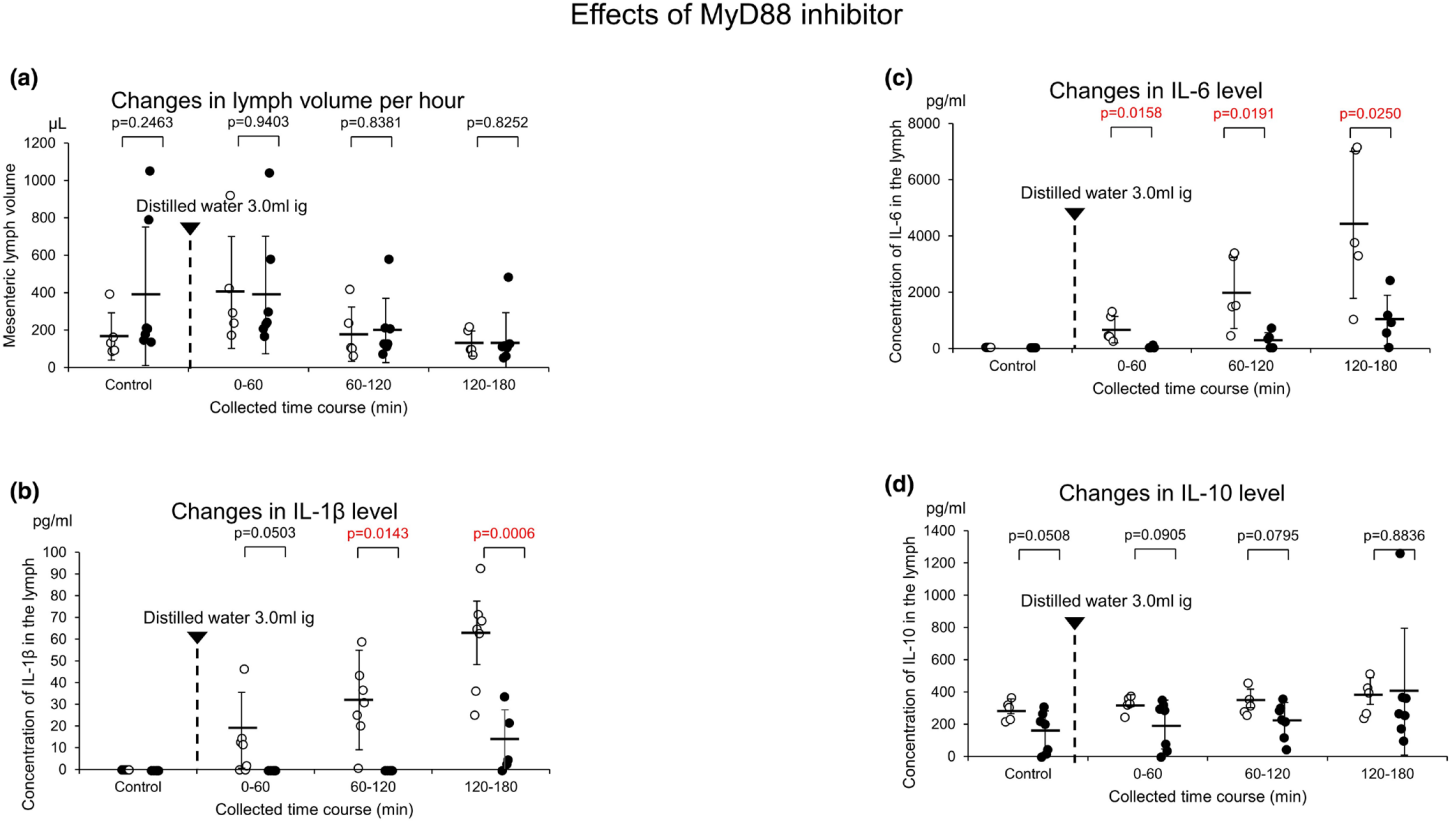
(a) Effects of water intake on changes in lymph volume collected over set intervals of 60 min in rat jejunum‐derived lymph vessels in the absence (white column) and presence of a MyD88 inhibitor (oblique line column). (b) Effects of water intake without (white column) and with a MyD88 inhibitor (oblique line column) on changes in the concentration of IL‐1β in the lymph. (c) Effects of water intake without (white column) and with a MyD88 inhibitor (oblique line column) on changes in the concentration of IL‐6 in the lymph. (d) Effects of water intake without (white column) and with a MyD88 inhibitor (oblique line column) on changes in the concentration of IL‐10 in the lymph. The error bars represent the SDs.

The pretreatment with MyD88 homodimerization inhibitory peptide (MyD88 inhibitor) reduced the lymph volume collected over set periods of 60 min from the jejunum‐originated lymph volume (Figure 4a; the control water intake without MyD88 inhibitor 166.0 ± 126.2 μL/60 min vs. the control water intake with MyD88 inhibitor 380.7 ± 370.3 μL/60 min, *p* = 0.2463 *n* = 5; 0–60 min water intake only 401.0 ± 299.0 μL/60 min vs. 0–60 min water intake with MyD88 inhibitor 387.1 ± 314.2 μL/60 min, *p* = 0.9403 *n* = 5; 60–120 min water intake only 178.0 ± 145.5 μL/60 min vs. 60–120 min water intake with MyD88 inhibitor 197.9 ± 171.7 μL/60 min, *p* = 0.8381 *n* = 5; 120–180 min water intake only 128.0 ± 67.2 μL/60 min vs. 120–180 min water intake with MyD88 inhibitor 144.3 ± 148.6 μL/60 min, *p* = 0.8252 *n* = 5). Thus, treatment with a MyD88 inhibitor caused no or little change in the lymph volume.

### 3.6 Pretreatment with a MyD88 inhibitor significantly decreased the water intake‐mediated release of IL‐1β and IL‐6 in the lymph

Pretreatment with the MyD88 inhibitor resulted in significant time‐dependent decreases in the water intake‐mediated release of IL‐1β and IL‐6 (Figure 4b, IL‐1β; the control water intake without the MyD88 inhibitor, 0.0 ± 0.0 pg/mL vs. the control water intake with the MyD88 inhibitor, 0.0 ± 0.0 pg/mL, NS *n* = 5; 0–60 min water intake only 18.0 ± 17.5 pg/mL vs. 0–60 min water intake with the MyD88 inhibitor, 0.0 ± 0.0 pg/mL *p* = 0.0503 *n* = 5; 60–120 min water intake only 32.0 ± 22.9 pg/mL vs. 60–120 min water intake with MyD88 inhibitor 0.0 ± 0.0 pg/mL, *p* = 00143 *n* = 5; 120–180 min water intake only, 63. 0 ± 14.6 pg/mL/60 min vs. 120–180 min water intake with the MyD88 inhibitor, 12.8 ± 14.6 pg./mL, *p* = 0.0006 *n* = 5).

The My88 inhibitor also significantly decreased the water intake‐mediated release of IL‐6 in a time‐dependent manner (Figure 4c, control water intake without the MyD88 inhibitor, 0.0 ± 0.0 pg/mL vs. the control water intake with MyD88 inhibitor, 0.0 ± 0.0 pg/mL/60 min, NS *n* = 5; the 0–60 min water intake only 670.0 ± 470.7 pg/mL vs. the water intake with MyD88 inhibitor 25.2 ± 38.8 pg/mL, *p* = 0.0158 *n* = 5; 60–120 min water intake only 1971.0 ± 1263.1 pg/mL/60 min vs. 60–120 min water intake with the MyD88 inhibitor 274.8 ± 289.8 pg/mL/60 min, *p* = 0.0191 *n* = 5; 120–180 min water intake only 4395. 0 ± 2613.6 pg/mL/60 min vs. 120–180 min water intake with MyD88 inhibitor 995.6 ± 895.2 pg/mL, *p* = 0.0250 *n* = 5).

### 3.7 Pretreatment with a MyD88 inhibitor caused no significant change in the water intake‐mediated release of IL‐10 in the lymph

In contrast, The treatment with the MyD88 Inhibitor produced no significant change in water intake release of IL‐10 (Figure 4d, the control water intake without MyD88 inhibitor 293.0 ± 63.3 pg/ mL vs. the control water intake with MyD88 inhibitor 153.7 ± 128.4 pg/mL, *p* = 0.0508 *n* = 5; 0–60 min water intake only 331.0 ± 51.2 pg/mL vs. the water intake with MyD88 inhibitor 197.9 ± 151.4 pg/mL, *p* = 0.0905 *n* = 5; 60–120 min water intake only 337.0 ± 79.6 pg/mL vs. 60–120 min water intake with MyD88 inhibitor 224.9 ± 109.4 pg/m, *p* = 0.0795 *n* = 5; 120–180 min water intake only 374.0 ± 115.3 pg/mL vs. 120–180 min water intake with MyD88 inhibitor 401.6 ± 0.399.7 pg/mL, *p* = 0.8836 *n* = 5).

## 4 DISCUSSION

The aims of this study were as follows: (1) to clarify whether water intake regulates jejunal mucosal immunity through the activity of inflammatory and anti‐inflammatory cytokines and (2) using a key substance in the signaling of macrophages, MyD88, we confirm whether the macrophages in the jejunal villi contribute to the regulation of jejunal mucosal immunity with cytokines.

The findings obtained in the present study are summarized as follows: (1) The intragastric administration of distilled water into rats increased jejunal‐originated mesenteric lymph flow approximately 0–60 min after administration. (2) Water intake led to the significant release and transport of IL‐1β and IL‐6 into the mesenteric lymph. However, compared with the changes to IL‐1β and IL‐6 concentration, the water intake did not significantly affect the concentration of IL‐10. The new findings with the inflammatory and anti‐inflammatory cytokines may demonstrate a big impact on gut mucosal immunity. Thus, the transient release of IL‐1β and IL‐6 into the mesenteric lymph may influence mucosal immune regulation or intestinal homeostasis. (3) Pretreatment with clodronate‐induced macrophage depletion significantly decreased the water intake‐mediated release and transport of IL‐1β and IL‐6 in the lymph. In contrast, the IL‐10 concentration in the lymph tended to have no or little change. (4) The immunoreactivities of the macrophage markers CD68, F4/80, and CD169 in the jejunal villi were significantly decreased by treatment with clodronate. (5) Pretreatment with a MyD88 inhibitor significantly decreased the water intake‐mediated releases of IL‐1β and IL‐6, but not the release of IL‐10.

The jejunum‐originated lymph flow rate may be related to the absorption time of water, which is very short. These findings may be consistent with experimental data from rats in which the highest volume of jejunum‐originated lymph was collected during the first 15 min after the administration of distilled water. In a previous study, we proposed that the specialized physiological properties of the jejunal microcirculation, that is, higher permeability for plasma albumin, may contribute mainly to the water intake‐mediated increase in jejunal‐originated mesenteric lymph flow. Thus, the leakage of albumin in the microcirculation may increase tissue osmotic pressure, which results in the accelerated transport of albumin‐binding water‐soluble substances, including distilled water into the lacteal vessels in the jejunal villi (Hayashi et al., 2021). In addition, the mechanical flushing effect from the increased lymph flow may be involved in mobilizing preformed cytokines.

Treatment with clodronate slightly reduced the mesenteric lymph flow, which may be related to clodronate‐induced macrophage depletion (Figure 3, 120–180 min). The depletion of macrophages may result in the release of numerous cellular substances of macrophages that interact with tissue components existing in the jejunal villi. This interaction has the potential to disrupt the movement of soluble substances through tissue fluid and lipoprotein‐ligated with albumin through the lamina propria, resulting in a modest clodronate‐mediated decrease of the lymph flow. The data are consistent with the finding that the MyD88 inhibitor significantly reduced the water‐intake‐mediated increase of lymph flow.

Clodronate‐induced macrophage depletion in jejunal villi also led to decreases in the concentrations of IL‐1β and IL‐6, but not IL‐10 in the lymph. Similarly, clodronate significantly reduced the cocoa butter‐mediated release of IL‐1β and IL‐6 (Arai et al., 2023). These findings may be related mainly to the activities of macrophages in the jejunal villi. In fact, as shown in Figure 3 the pretreatment with clodronate immunohistochemically confirmed the disappearance of macrophages in the lamina propria of the jejunal villi.

Based on these findings, to confirm the involvement of macrophages, we investigated the effects of the key molecular signaling substance in macrophages, MyD88, on the water intake‐mediated release of inflammatory and anti‐inflammatory cytokines. In fact, pretreatment with the MyD88 inhibitor significantly decreased the water intake ‐mediated releases of IL‐1β and IL‐6 in a time‐dependent manner. In contrast, the treatment with MyD88 inhibitor produced no significant change in water intake or the release of IL‐10. The IL‐10 can originate from multiple cell types, including regulatory T cells, dendric cells, and epithelial population so that the lack of water intake‐mediated IL‐10 release may reflect cell‐type‐specific regulation or temporal differences in cytokine release dynamics. However, clodronate inhibited IL‐1β but not IL‐6, whereas the MyD88 inhibitor blocked both cytokines in the lymph. These findings suggest that IL‐6 may be released from the non‐macrophage, MyD88‐dependent cells, for example, dendritic cells, regulatory T cells, and epithelial cells (Banias & Ciminelli, 2016; Latz et al., 2013; Saraiva & O'Garra, 2010). In particular, dendric cells residing in the lamina propria are known to express MyD88‐dependent Toll‐like receptor pathways and are capable of IL‐6 and IL‐1 family cytokines. Similarly, epithelial cell subsets, including Patheth cells, are increasingly recognized as active contributors to mucosal immune regulation through cytokines and antimicrobial peptide secretion. However, macrophage depletion may also indirectly affect the activity of neighboring immune and epithelial cells within the jejunal villi microenvironment. Thus, potentially secondary effects on dendric cell function or epithelial signaling would provide a more balanced interpretation and strengthen the overall mechanistic framework. The detailed contributions of dendritic cells, macrophages, and the other cells in the jejunum to the water induced regulation of mucosal immunity will need to be investigated in the future.

These findings also suggest that macrophages in the jejunal villi are related to water intake‐mediated increases in the concentrations of the inflammatory cytokines, IL‐1β and IL‐6, but not IL‐10. IL‐10 is stored in not only the macrophages but also regulatory T cells (Tregs) and helper T2 cells (Th2 cells) distributed in the lamina propria of jejunal villi (Saraiva & O'Garra, 2010; Schnell et al., 2021). In addition, the contribution of M1 or M2 macrophages to the obtained data with clodronate is not clear. Further investigations are needed to define key roles of M1 or M2 macrophages in the water intake‐mediated release of IL‐1β and IL‐6.

**FIGURE 5:**
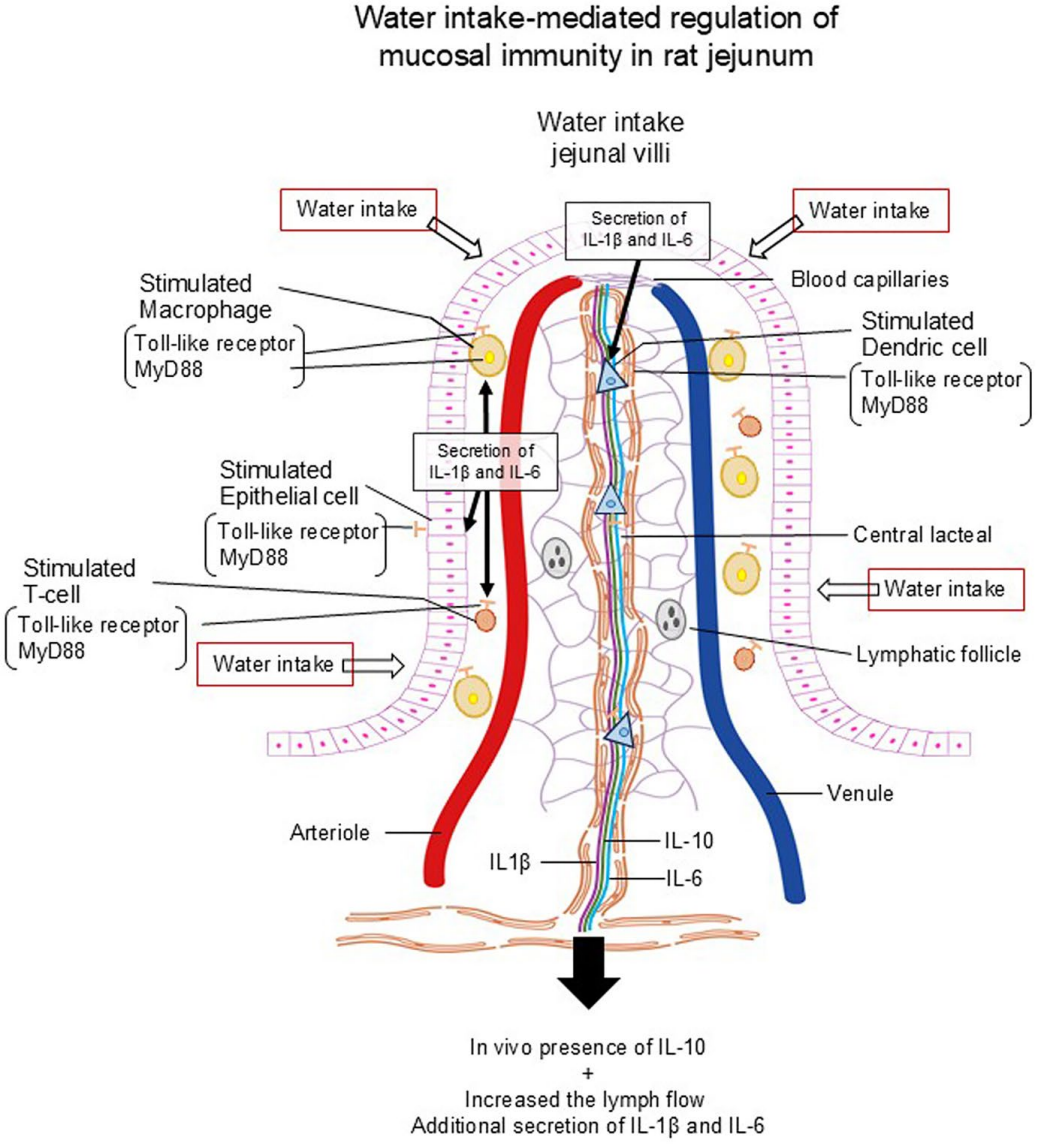
The graphical abstract shows the conclusion in this study. Microvilli, Lymphatic vessel, Dendric cell, Helper T cell, Nerve fiber, Submucosa.

In conclusion (Figure 5), the contribution of water intake maintaining health care was clarified through in vivo experiments in rats in the present study. The present study is also the first to report the acute effects of water intake on the release and transport of inflammatory and anti‐inflammatory substances in male rat jejunal‐originated lymph. In addition, this study demonstrated that the macrophages located in the jejunal villi contributed to the water intake‐mediated release of IL‐1β and IL‐6. In accordance with these findings, the macrophages in the jejunal villi are recognized as one of the sources of IL‐1β and IL‐6 (Banias & Ciminelli, 2016; Wang & Li, 2020; Zhang et al., 2019). Thus, water intake regulates gut mucosal immunity through the activation of the release and transport of IL‐1β, IL‐6, and IL‐10, which are inflammatory and anti‐inflammatory cytokines, into the mesenteric lymph vessels.

However, in the present study we have incompletely discussed the specific roles of IL‐1β and IL‐6 in mucosal immunity and homeostasis. In addition, the limitations of the study are the lack of data on other cell types, for example, dendritic cells, regulatory T cells, and epithelial cells. Further investigation will be, in the future, needed to clarify the contribution of the cells in mucosal immunity. In addition, several findings in the study are described as trends that do not reach statistical significance, so that we will also, in the future, conduct additional experiments with a larger sample size to obtain clearer data.

## ARRIVE GUIDELINES

The study is reported in accordance with ARRIVE guidelines.

## AUTHOR CONTRIBUTIONS

**Norika Kuneshita:** Conceptualization; data curation; formal analysis; investigation; methodology; project administration; validation. **Yuki Sakai:** Data curation; formal analysis; investigation; methodology. **Moyuru Hayashi:** Conceptualization; data curation; formal analysis; investigation; methodology; project administration; validation. **Tomomi Watanabe‐Asaka:** Conceptualization; data curation; formal analysis; investigation; methodology; validation. **Daisuke Maejima:** Conceptualization; data curation; formal analysis; investigation; methodology; project administration. **Yoshiko Kawai:** Conceptualization; data curation; formal analysis; investigation; methodology; project administration; supervision; validation. **Toshio Ohhashi:** Conceptualization; data curation; formal analysis; funding acquisition; investigation; methodology; project administration; supervision; validation.

## FUNDING INFORMATION

The donation (2019–2023) of BOURBON, Co., Ltd., Kashiwa Zaki, Niigata, Japan.

## CONFLICT OF INTEREST STATEMENT

The authors declare no conflicts of interest.

## ETHICS STATEMENT

This study and all the experimental protocols were approved by the Institutional Animal Care and Use Committee of Shinshu University (No. 023017, 1st April, 2019).

## CONSENT

All the authors approved the final version of the manuscript and consent to its publication manuscript.

## Data Availability

All relevant data are available from the corresponding author, Toshio Ohhashi, on request.
